# Routine postoperative nursing management

**Published:** 2016

**Authors:** Ebby Adekhera

**Affiliations:** Nursing Officer: Sabatia Eye Hospital, Wodanga, Kenya.

**Figure:**
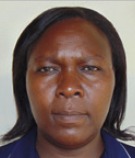
Ebby Adekhera

The nursing process is a systematic, scientific approach to managing a range of patients. This article explains how the nursing process can be applied when caring for cataract patients who have been admitted.

The nursing process consists of five phases of management:

- Assessment
- Diagnosis
- Planning
- Implementation
- Evaluation.

## Assessment

Assessment is done by using effective communication and observational skills to carry out a complete and holistic nursing assessment of every patient's needs. An actual or potential problem with the patient (i.e. pain, or an infection following cataract surgery) may be discovered.

**Figure 1.**
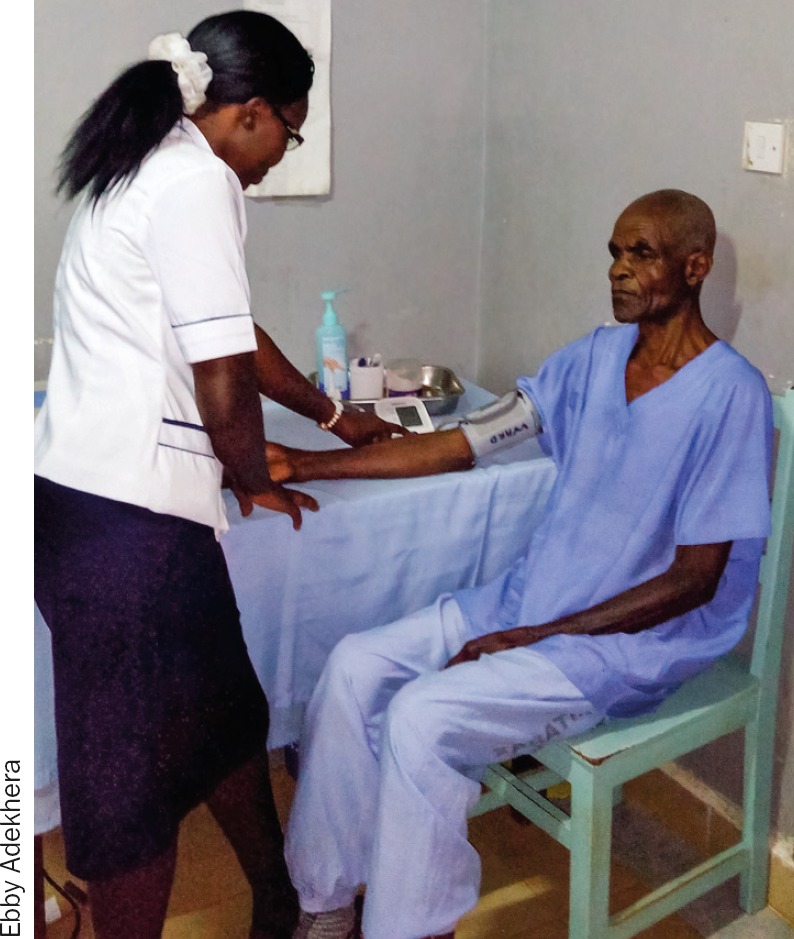
Measuring vital signs before surgery.

Before surgery, take a history of the patient and obtain their baseline blood pressure and pulse (Figure 1).

After surgery, look at the patient's facial expression to determine if she or he is in pain and ask the patient how she or he is feeling. Measure vital signs (pulse and blood pressure).

From the first day after surgery (day 1), carry out an eye examination to look at visual acuity, the state of the wound, the conjunctiva, the cornea, the anterior chamber, the pupil and the position of the intraocular lens. Observe the patient for any signs of infection (redness, swelling or discharge), ask about pain and treat or refer the patient as appropriate.

At later follow-up visits, measure visual acuity to assess the need for refraction and spectacle correction, in collaboration with the patient.

## Diagnosis

After the assessment phase, determine and prioritise the patient's nursing needs, **from their basic health needs to their eye care.** Most importantly, be on the lookout for signs of complications: most commonly worsening sight, increasing pain, redness, swelling or discharge.

## Planning

With the patient's agreement, consider each of the problems identified, plan to manage them according to priority and seta measurable goal. For example, for pain, plan to give analgesics and reassure the patient. If there are signs of a postoperative complication, plan to either treat the complication or make a referral, depending on the suspected complication.

## Implementation

Next, record the methods by which the goals will be achieved in a clear format that all can understand. For example, record the time and dose when analgesics are administered. It is important to know the appropriate dose and be able to identify any side effects.

## Evaluation

This is a continuous process in which we look at the initial and the present situation, compare the two and evaluate progress towards the goals identified in the previous stages. If progress towards the goal is slow or if regression has occurred, change the plan of care accordingly. If the goal has been achieved, then the care can cease. For example, if a patient is relieved of pain, stop the analgesics. If not, adjust the plan and change to another form of management, depending on the cause of the pain.

